# Multifunctional Application of Electrospun Fiber

**DOI:** 10.3390/polym17182519

**Published:** 2025-09-18

**Authors:** Rui Zhao

**Affiliations:** Faculty of Chemistry, Northeast Normal University, Changchun 130024, China; zhaor814@nenu.edu.cn

## 1. Introduction

Nanofibers have become an important development in materials science and engineering fields, owing to their exceptional material properties, such as their small size, high modulus, wide range of applications, etc. [1]. The developed fabrication techniques for nanofibers are diverse, including template synthesis, self-assembly, meltblowing, stretching, phase separation, and electrospinning [2,3,4,5]. Among these techniques, electrospinning stands out for its significant comprehensive advantages and has emerged as a preferred choice for both research and industrialization. Its exceptional material diversity is evident by its ability to process almost all spinnable polymers (synthetic/natural polymers, ceramic precursors, composite materials, etc.) while precisely controlling fiber diameter, morphology, orientation, porosity, and membrane thickness by adjusting solution parameters, process parameters, and environmental conditions [6,7,8]. Accordingly, this method ultimately enables the effective fabrication of organic, inorganic, and organic/inorganic composite nanofibers. Moreover, electrospinning exhibits notable cost-effectiveness, characterized by low equipment investment and operational costs, as well as high material utilization efficiency, making it highly competitive for industrial applications [9,10].

Compared to traditional fiber materials, electrospun nanofibers exhibit unique appeal because of their capacity for precise material customization according to broad and diverse application requirements. This has revealed significant application potential, attracting substantial attention and sustained investment in research from material scientists and engineers worldwide. The distinctive features of electrospun nanofibers are the key to their popularity: an extremely high specific surface area provides a number of active interfaces for adsorption, catalytic reactions, and sensing; a highly tunable fiber structure (from nano- to micro-scale) and surface morphology (e.g., smooth, rough, porous, core–shell, heterogeneous structures) enable the accurate regulation of substance transport, selective separation, and cellular behavior [11,12,13]; the unique morphological characteristics allow researchers to design and fabricate ultra-lightweight, highly porous materials with fine structural controllability and multifunctional integration [14,15]. Meanwhile, these materials also exhibit good mechanical properties, outstanding chemical stability, and specific thermal properties. As a result, electrospun nanofibers are widely used in biomedical applications, environmental and energy technologies, electronics and smart wearables, and safety and protection fields, continuously driving innovation and application development in nanofiber materials.

Despite significant progress, several challenges remain in comprehensively realizing the industrial application of electrospun fibers. In terms of material performance, most electrospun fibers rely on single-polymer substrates, making it difficult to balance functional characteristics, mechanical strength, biocompatibility, and conductivity. For example, in the biomedical field, high tensile strength and degradability often present a contradiction. Additionally, their dynamic responsiveness to complex environments is weak. Under conditions of drastic humidity or temperature changes, fiber structures are prone to swelling or embrittlement, limiting their use in smart wearables and protection in extreme environments. In terms of production efficiency, conventional single-nozzle spinning methods suffer from low productivity, susceptibility to clogging, and difficulties in parameter control. On the other hand, there is also a disconnect between clinical adaptation and industrial transformation. In medical implant applications, the degradation rate of fiber scaffolds often fails to match the pace of tissue regeneration, leading to premature failure or inflammatory responses. In functional device integration, poor interfacial compatibility between nanofibers and electronic components often results in an insufficient stability of self-powered and sensing-based smart devices, generally generating short service lives that fail to meet practical application requirements. These existing issues indicate that there are still numerous research gaps in the field of electrospun nanofibers, necessitating continuous and in-depth exploration by researchers. The studies in this Special Issue address the limitations of electrospun nanofibers in various fields by proposing practical solutions, thereby advancing the development of electrospinning technology and nanofiber materials.

## 2. Overview of Published Articles

The collective contributions in this Special Issue reflect the applications and advancements of electrospun nanofibers across multiple fields, e.g., biomedicine and drug delivery, tissue engineering and regenerative medicine, environmental remediation and adsorption materials, air filtration and protection, antibacterial and antifungal applications, flexible electronics and energy devices, etc. The following section provides a detailed introduction to the relevant research:

Luca Éva Uhljar et al. [16] successfully developed an orally disintegrating drug delivery system based on PVP nanofibers containing diclofenac sodium, which were prepared from dual-nozzle electrospinning combined with 3D printing. The formulation exhibits ultra-fast disintegration, immediate release, high drug loading efficiency, good stability, and biocompatibility, presenting a highly promising oral dispersible drug delivery system. Additionally, a novel in vitro dissolution method (“AS-to-FaSSGF”) was developed to provide a more comprehensive understanding of drug dissolution in the digestive tract. This study presents a new process to obtain nanofibers via combining electrospinning and 3D printing. Moreover, the prepared materials are a promising drug release system with application potential.

Daniela Anahí Sánchez-Téllez et al. [17] developed sol–gel siloxane-crosslinked polyvinyl alcohol (PVA)–hyaluronan (HA) electrospun nanofibrous mats. This crosslinking effectively inhibited the hydrolytic degradation and metabolic breakdown of HA while simultaneously providing structural support to cells and enhancing the intrinsic osteogenic potential of HA. The nanofiber scaffold with high PVA content supported three-dimensional spherical osteoblast morphology (indicative of tissue growth), as well as sustained cell adhesion, proliferation, differentiation, and calcium deposition over 21 days independent of exogenous growth factors. The nanofiber scaffold also exhibited a controlled degradation rate and enhanced osteogenic performance. Siloxane crosslinking overcomes its application limitations of electrospinning HA, making this a breakthrough material for bone tissue regeneration engineering.

Fatma Sude Cetin et al. [18] successfully fabricated chitosan/polyethylene oxide/bacterial cellulose (CS/PEO/BC) composite nanofibers via electrospinning. CS/PEO/BC nanofibers showed a significant improvement in tensile strength (approximately 3.4 MPa, about 59% higher than chitosan/polyethylene oxide nanofibers), ultra-high water absorption capacity exceeding 200%, excellent biocompatibility (99.26% cell viability on day 7), and good biodegradability. More importantly, CS/PEO/BC nanofibers exhibited good antimicrobial activity against Escherichia coli and Staphylococcus aureus. Therefore, these nanofibers represent highly promising materials for advanced biomedical applications, particularly in tissue engineering.

Leire Murillo et al. [19] successfully constructed an antifungal functional material through a two-step process, involving electrospinning polyethylene (PEO)/chitosan (CS) nanofibers (124 ± 36 nm) followed by dip-coating for an in situ synthesis of silver nanoparticles (AgNPs). AgNPs increased the fiber diameter to 330 ± 106 nm, reduced roughness to 38.9 nm, and increased the contact angle from 23.4° to 97.7° (enhanced hydrophobicity). PEO/CS/AgNPs achieved a 39.7% inhibition rate after 13 days of fungal (Pleurotus ostreatus) exposure and formed significant inhibition zones after 10 days. The external loading of AgNPs (content 10.23%) and the synergistic antimicrobial mechanism provide a long-lasting antifungal solution for medical devices and food packaging.

Chunling Zheng et al. [20] designed a β-cyclodextrin/polyacrylic acid (β-CD/PA) composite nanofibrous membrane from the electrospinning process. Through host–guest inclusion and electrostatic synergy, the adsorption capacities of β-CD/PA nanofiber adsorbents toward basic red 9, basic red 14, basic red 46, basic blue 9, basic yellow 19, and basic yellow 28 were 86.7 mg/g, 21.5 mg/g, 18.9 mg/g, 44.5 mg/g, 116.5 mg/g, and 155.2 mg/g, respectively. The effects of different initial concentrations and pH values were also used to study adsorption performance. It performed excellently across a wide concentration range and at neutral pH. In addition, adsorption was followed pseudo-second-order kinetics, and the nanofiber adsorbent was easily recyclable and renewable, making it a promising renewable adsorbent for dye wastewater treatment.

Xingyu Fu et al. [21] designed quaternary ammonium group-functionalized electrospun nanofibers guided by theoretical calculations. They achieved an ultra-high adsorption capacity for free bilirubin (818.9 mg/g) and an adsorption capacity for albumin-bound bilirubin of 163.7 mg/g (removal rate > 90%). Their dynamic adsorption performance and blood compatibility are significantly superior to commercial activated carbon and resins. This study demonstrates the effectiveness of the theory-driven design method to develop electrospun nanofiber adsorbents and provides an efficient and safe adsorbent paradigm for blood toxin hemoperfusion treatment.

Huanliang Liu et al. [22] prepared polysulfone (PSF) fibrous membranes (diameter: ~1.17 μm) via one-step fast electrospinning using a N, N-dimethylformamide/tetrahydrofuran as the solvent in a volume ratio of 3:1. Leveraging their highly porous structure, excellent hydrophobicity (contact angle > 110°), and mechanical toughness (tensile strength of 1.14 MPa, elongation at break of 116.6%), they achieved ultra-high particulate matter removal performance. The filtration efficiencies for PM2.5 and PM1.0 99.6% and 99.2%, respectively, had a low pressure drop of ~65 Pa. The PSF fibrous membranes maintained these filtration efficiencies over 28 days without decay. The simple preparation process, low cost, and high PM removal efficiency make the PSF fibrous membrane a highly promising replacement for commercial masks and air conditioning filters in various industrial filtration applications.

Sujin Ryu et al. [23] constructed a dual-layer biodegradable membrane which was integrated from electrospun cellulose acetate nanofibers (first layer) deposited on the following layer via electrospinning and zeolitic imidazolate framework (ZIF-8)-coated cotton fabric (second layer). The first layer was used to filtrate particulate matter (PM), and the second layer was used to remove toxic gases. At the optimal thickness of the electrospun fiber layer, the filtration membrane achieved 93.1% PM filtration efficiency, an ultra-low-pressure drop of 14.2 Pa, and a quality factor of 18.8 × 10^−2^ Pa^−1^; meanwhile, it showed toxic gas adsorption removal rates > 99% for H_2_S, formaldehyde, and NH_3_. The dual-layer filtration membrane was also environmentally friendly and exhibited a 62.5% biodegradation rate in 45 days. This study provides an innovative material with a multilayered structure for personal protective equipment that combines efficient filtration, gas purification, and full lifecycle sustainability.

Baodong Sun et al. [24] optimized inorganic NiMoO4 nanofibers via electrospinning and high-temperature calcination at 500 °C. Their unique nanofiber structure could effectively suppress powder agglomeration, which enabled an ultra-high specific capacitance of 1947 F/g in supercapacitors and a capacity retention rate of 82.4% after 3000 cycles. Their efficient charge transfer and ion diffusion characteristics make them the ideal electrode material for high-performance portable energy storage devices.

Popat Mohite et al. [25] discussed the characteristics of gelatin (Ge) and chitosan (CH), their roles in formulation strategies, and characterization techniques for electrospun fibers, aiming to provide a comprehensive overview of the potential of Ge/CH-based nanofibers in drug delivery and biomedical applications. The Ge/CH-based electrospun nanofiber drug delivery system, leveraging its biomimetic ECM structure, natural biocompatibility, and degradability, shows breakthrough potential in orthopedic repair and wound healing. Optimizing electrospinning processes and functionalization (e.g., with targeting ligands/stimuli-responsive elements) can overcome challenges in fiber structure control, drug-loading stability, and clinical translation, offering a new generation of biomedical solutions for precise delivery and multi-drug combination therapy. This review also prospects the full potential of these nanofibers for managing a range of health complications.

Emanuele Alberto Slejko et al. [26] systematically summarized the preparation, properties, and applications of poly (3,4-ethylenedioxythiophene) (PEDOT)-based nanofibers. By analyzing recent advances in their synthesis, functionalization, and post-processing, the article elaborates on how to fully exploit the potential of this material in next-generation electronics and functional devices. Electrospun PEDOT nanofibers, achieved via coaxial spinning/carrier polymer strategies, offered tunable optoelectronic properties and flexible structures, overcoming processing challenges posed by rigid molecular chains. They provided a core material platform for wearable electronics and biosensing. Future studies need to address industrial-scale stability and roll-to-roll continuous production to deepen bioelectronic integration applications. The review also identifies current challenges and future directions, proposing feasible paths to address the issues of scalability, reproducibility, stability, and device integration.

Yaoyao Yang et al. [27] discussed in detail the application and development prospects of electrospinning technology in drug delivery for cancer treatment. Leveraging exceptional material compatibility, this technology could flexibly combine biocompatible organic compounds, drug molecules, and gene fragments to construct functionalized nanofiber carriers. Compared to oral and implantable drug delivery, electrospun carriers enabled localized, sustained, and controllable drug release, significantly enhancing tumor targeting. Key advantages included synergistic therapeutic mechanisms, multi-modal integration capability, and customization for the pathological microenvironment. These characteristics make electrospinning a breakthrough strategy meeting the demands of personalized cancer therapy, providing effective technical support for next-generation intelligent anti-tumor systems.

## 3. Summary and Future Outlook

These twelve articles collectively elucidate the current trends in electrospun fiber research. Through precise control of fiber morphology, composition, and functionalization, the high specific surface area and porous structure of the nanofibers are utilized to enhance performance, e.g., improving adsorption/drug-loading capacity, achieving low-resistance filtration, and utilizing biomimetic topological structures to promote cell proliferation and tissue regeneration. Synergistic composite effects overcome the limitations of single functionality. These groundbreaking advances collectively demonstrate that electrospinning, by precisely regulating fiber structure and function, has become an important platform to drive innovation across multiple fields. Each study provides a unique perspective, deepening the scientific and engineering understanding of the design and fabrication processes for next-generation electrospun materials. However, related research still needs to further address challenges such as stability in scaled-up production, compliance for clinical translation, and design for environmental degradation.

The future development of electrospinning technology should not only focus on fundamental research, such as new theories, materials, technologies, devices, and applications, but also expand the application scope of electrospun fibers. According to statistics, the demand for electrospun nanofibers is highest in filtration materials among various applications. Expanding the market for electrospun nanofibers in other fields requires the collective efforts of practitioners in the electrospinning industry. It is hoped that through a collaborative industry–academia–research model of “scientific breakthroughs in universities–industrialization practices in enterprises”, more electrospun nanofiber materials can move from the laboratory to the marketplace, promoting the true realization of industrial value for electrospun nanofibers. Additionally, future research should focus on integrating electrospinning with artificial intelligence (AI), using AI to optimize various parameters and conditions and taking advantage of material genome technology to ultimately enhance production efficiency, unlock material properties, and overcome industrialization bottlenecks. In this Special Issue, we aim to attract broader participation in electrospinning research.
